# Prevalence of Gastrointestinal Symptoms and Clinical Outcomes in Hospitalized Coronavirus Disease 2019 Patients: A Single-Center Study from Turkey

**DOI:** 10.5152/tjg.2022.21484

**Published:** 2022-11-01

**Authors:** Süleyman Dolu, Göksel Bengi, Vildan Avkan-Oğuz, Kemal Can Tertemiz, Naciye Sinem Gezer, Sema Alp Çavuş, Aylin Özgen Alpaydın, Ziya Kuruüzüm, Begüm Ergan, Can Sevinç, Gökçen Ömeroğlu Şimsek, Oğuz Kılınç, Murat Örmen, Arzu Sayıner, Işıl Somalı, Caner Çavdar, Gerçek Can, Tevfik Demir, Mesut Akarsu, Yusuf Savran, Müjde Soytürk

**Affiliations:** 1 Department of Gastroenterology, Dokuz Eylül University Faculty of Medicine, İzmir, Turkey; 2 Department of Infectious Diseases and Clinical Microbiology, Dokuz Eylül University Faculty of Medicine, İzmir, Turkey; 3 Department of Pulmonary Diseases, Dokuz Eylül University Faculty of Medicine, İzmir, Turkey; 4 Department of Radiology, Dokuz Eylül University Faculty of Medicine, İzmir, Turkey; 5 Department of Biochemistry, Dokuz Eylül University Faculty of Medicine, İzmir, Turkey; 6 Department of Medical Microbiology, Dokuz Eylül University Faculty of Medicine, İzmir, Turkey; 7 Department of Medical Oncology, Dokuz Eylül University Faculty of Medicine, İzmir, Turkey; 8 Department of Nephrology, Dokuz Eylül University Faculty of Medicine, İzmir, Turkey; 9 Department of Rheumatology, Dokuz Eylül University Faculty of Medicine, İzmir, Turkey; 10 Department of Endocrinology, Dokuz Eylül University Faculty of Medicine, İzmir, Turkey; 11 Department of Internal Medicine and Medical Intensive Care, Dokuz Eylül University Faculty of Medicine, İzmir, Turkey

**Keywords:** COVID-19, gastrointestinal symptom, outcomes, prognosis, mortality

## Abstract

**Background:**

In patients with coronavirus disease 2019, the gastrointestinal symptoms have been reported increasingly in addition to the respiratory system symptoms. The studies show that the prevalence of gastrointestinal system symptoms and how the gastrointestinal system contributes to the severity and prognosis of the disease is still not clear. This study aims to find the prevalence of gastrointestinal symptoms and the correlation between the gastrointestinal symptoms and the clinical results in hospitalized patients diagnosed with coronavirus disease 2019.

**Methods:**

This study retrospectively analyzes patients diagnosed with coronavirus disease 2019 and hospitalized in the pandemic unit between March 2020 and August 2020 and compares their demographic and clinical characteristics, laboratory and radiologic findings, coronavirus disease 2019 treatments received, the clinical course of the disease, and the gastrointestinal symptoms.

**Results:**

In our study, we included 322 patients diagnosed with coronavirus disease 2019 and hospitalized; 39 patients (12.1%) were admitted to the hospital with at least one gastrointestinal symptom (nausea and vomiting, diarrhea, abdominal pain, and the loss of taste). Nausea and vomiting are the most common gastrointestinal symptoms with a prevalence of 7.1%, followed by diarrhea with 2.8%, the loss of taste with 2.2%, and abdominal pain with 1.5%. The mean age and D-dimer levels of the patients showing gastrointestinal symptoms were lower than those who did not have any gastrointestinal symptoms. We did not find a significant correlation between the presence of the gastrointestinal symptoms and the severity of the disease, treatment received, risk of acute respiratory distress syndrome and septic shock, admission to the intensive care unit, the need for mechanical ventilation, the mortality rate or the length of hospitalization in the medical floor or the intensive care unit.

**Conclusion:**

In this study, we observed that 12.1% of coronavirus disease 2019 patients apply to the hospital due to gastrointestinal symptoms. Furthermore, the gastrointestinal symptoms do not seem to affect the severity and the course of the disease, it is important to identify coronavirus disease 2019 patients showing unusual symptoms such as the gastrointestinal symptoms at an early stage to protect healthcare professionals from infection risk.

## Introduction

The novel severe acute respiratory syndrome coronavirus 2 (SARS-CoV-2), also known as novel coronavirus disease 2019 (COVID-19), is a single-stranded ribonucleic acid (RNA) virus that has spread around the world since December 2019.^1,2^ As of May 25, 2021, 167 252 150 people were infected worldwide and 3 467 663 people died while 5 186 487 people were infected and 46 268 people died in Turkey.^3^

It has been proven that COVID-19 is transmitted from person to person through close contact and respiratory droplets.^4^ It has also been shown that people can be infected via fecal-oral route, causing gastrointestinal (GI) infections after the virus is cleared from the upper respiratory tract.^5^ The manifestation in symptomatic COVID-19 patients varies from mild respiratory system infections to severe pneumonia and even acute respiratory distress syndrome (ARDS) and multiple organ failure.^1,6^ The common COVID-19 symptoms include fever, cough, fatigue, myalgia, and dyspnea, but the GI symptoms such as diarrhea, nausea, vomiting, anorexia, and abdominal pain can also be observed.^4,7^ The study results vary in terms of the prevalence of GI symptoms.^8^ Although studies in this field are heterogeneous, the prevalence of GI symptoms accompanying COVID-19 has been reported to be 3%-79%. In addition, the most common GI symptom and its prevalence vary in this patient group.^9^

It is still not clear how GI involvement contributes to the severity and prognosis of the disease. Some studies report that GI involvement increases the severity of the disease, and such patients are more prone to develop ARDS, and the need for mechanical ventilation and intensive care is higher.^1^ Nevertheless, some studies show that complication rates and clinical prognosis are similar in patients with and without GI symptoms.^10,11^

In an attempt to present our country’s data, this study aims to investigate the prevalence of GI symptoms at admission and the prognosis of patients with GI complaints during the follow-up period in patients hospitalized with COVID-19 diagnosis.

## MATERIALS AND METHODS

### Study Design and Participants

This retrospective study analyzes the data of patients diagnosed with COVID-19 and hospitalized in the pandemic unit of Dokuz Eylül University Hospital between March 2020 and August 2020. The study involved patients admitted to the hospital with a COVID-19 diagnosis, either possible or definitive according to the Republic of Turkish Ministry of Health guidelines, version March 2020.^12^ In our hospital, the multidisciplinary council comprised at least 1 pulmonologist, infectious diseases specialist, microbiologist, radiologist, and internal medicine specialist examined patients’ laboratory, clinical, and radiologic findings to confirm the COVID-19 diagnosis (possible or definitive). According to these evaluations, patients with symptoms such as fever, cough, dyspnea, tachypnea, hypoxemia, hypotension, common radiological findings on chest computed tomography (CT), and changes in consciousness in addition to acute respiratory tract infection that developed in the last 14 days were hospitalized and followed up in the pandemic service.

All hospitalized patients received standard treatment protocol according to the Republic of Turkish Ministry of Health guidelines, version March 2020.^12^

### Data Collection

We obtained information about the age, gender, smoking history, occupation, chronic diseases, complaints, and medication of the patients admitted to the pandemic unit from the medical records kept at the time of admission. The patients were tested for SARS-CoV-2 RNA by real-time reverse-transcription polymerase chain reaction (RT-PCR) using their nasopharyngeal swab samples taken at the time of admission, and the other respiratory tract virus agents were excluded.

The thorax area of all hospitalized patients was scanned by CT at least one time. A 64-channel multidetector CT scanner (Brilliance, Philips Medical Systems, Holland) reserved for COVID-19-suspected patients was used for imaging. Computed tomography examinations were performed without an intravenous contrast medium. The computed tomography imaging protocol was as follows: 120 kVp, 80 mA, slice thickness 1.5 mm, and high-spatial-frequency reconstruction algorithm (bone algorithm). Chest CT examinations were evaluated by a board-certificated radiologist with 14 years of experience. For CT diagnosis of COVID-19 associated pneumonia, CT scans were classified as classical/probable COVID-19, indeterminate COVID-19, non-COVID-19, and normal according to the recommendation of the British Thoracic Society.^13^

Patients who complained about abdominal pain, loss of taste, nausea–vomiting, or diarrhea at their admission to the hospital were defined as the patient group with GI symptoms. Diarrhea was defined as defecating more than 3 times a day or the softening of the stool into a more liquid form. Furthermore, stool culture and fecal parasite tests were conducted for the patients with diarrhea to eliminate other causes of diarrhea, and their recent antibiotic use was also taken into consideration.

In addition, patient’s laboratory test results for hemogram, neutrophil-lymphocyte ratio, D-dimer, creatinine, alanine aminotransferase (ALT), aspartate aminotransferase (AST), albumin, lactate dehydrogenase (LDH), troponin, ferritin, C-reactive protein (CRP), and procalcitonin on their first examination and the third and seventh day of admission were also recorded.

During the follow-ups, the severity of the disease, risk of ARDS and septic shock, the need for mechanical ventilator and intensive care, mortality, and the length of stay in the intensive care unit and medical floor were recorded. The severity of COVID-19 was defined according to the diagnosis and treatment protocol for novel coronavirus pneumonia (version 7) released by the National Health Commission and State Administration of Traditional Chinese Medicine: (1) mild: symptoms are mild, and there are no signs of pneumonia on imaging; (2) moderate: fever, respiratory symptoms with radiological evidence of pneumonia; (3) severe: meeting one or more of the following criteria: respiratory distress, the breathing rate of 30 breaths a minute, hypoxia, oxygen saturation (SpO_2_) ≤93% (at rest); and (4) critical: meeting any of the following criteria: respiratory failure requiring mechanical ventilation, shock, or multiple organ dysfunction requiring intensive care unit monitoring and treatment.^14^

## Outcome Data

The patients were divided into 2 groups based on the presence of GI symptoms. We compared the correlation between the demographic characteristics, clinical characteristics, laboratory and radiologic findings, COVID-19 treatment they received, the clinical course of the disease, and the presence of GI symptoms.

This study was conducted with the approval of the Non-Invasive Clinical Research Ethics Committee of Dokuz Eylül University (Date: October 08, 2020, No: 2022/18-22) and the Ministry of Health Scientific Research Approval Department (Form name: 2020-07-2T12_36_52). The study was conducted according to the criteria set by the Declaration of Helsinki and patients’ information were kept confidential.

### Statistical Analysis

The descriptive findings were presented with mean ± standard deviation (SD) or median (0.25-0.75 percentile) for the continuous variables and with frequency and percentage for the categorical variables. The normality assumptions were controlled by the Shapiro–Wilk test. Categorical data were compared with Pearson chi-square and Fisher’s exact test. Mann–Whitney *U* test and independent *t*-test were used for the analysis of non-normally and normally distributed continuous data, respectively. A two-sided *P* value less than .05 was considered statistically significant. All analyses were made using IBM SPSS Statistics for Windows, Version 23.0 (IBM Corp., Armonk, NY, USA).

## Results

This study included 322 patients diagnosed with COVID-19 and hospitalized in the pandemic unit. The mean age of patients in this study was 61.02 ± 20.63 years, and 159 (49.4%) of them were male. Thirty-nine patients (12.1%) had at least 1 GI symptom alone or with other GI symptoms. The prevalence of GI symptoms was as follows respectively: nausea–vomiting (n = 23, 7.1%), diarrhea (n = 9, 2.8%), the loss of taste (n = 7, 2.2%), and abdominal pain (n = 5, 1.5%). Seven (2.2%) patients only had diarrhea, 20 (6.2%) patients only felt nausea or vomiting, and 7 (2.2%) patients complained only about the loss of taste. Apart from these, abdominal pain was accompanied by nausea and vomiting in 3 patients and diarrhea in 2 patients. In our study, the mean age of patients with GI symptoms was 53.72 ± 19.71 years, which was found to be lower than the mean age of those without the GI symptoms (*P* = .018). Gastrointestinal symptoms were less common in patients with at least 1 chronic disease or a history of chronic medication use (*P* = .005; *P* = .029). When patients’ complaints at their first arrival were evaluated, it was seen that patients with GI symptoms complained about the loss of smell more frequently than those not showing any GI symptoms (*P* < .001); however, there was no such correlation with other symptoms (Table 1).

In our study, 186 (57.8%) patients were RT-PCR positive and 136 (42.2%) patients were RT-PCR negative using their nasopharyngeal swab samples. Gastrointestinal complaints were less common in patients with positive RT-PCR (*P* = .024). D-dimer was found to be lower in patients with GI symptoms (*P* = .043). The other laboratory results and radiological findings were similar in patients with or without GI symptoms (Table 2). There was no correlation between the GI symptoms and the patient’s laboratory test results on the third and seventh days of admission.

The most frequent treatment administered for COVID-19 was hydroxychloroquine treatment, and no significant correlation was found between the GI symptoms and the treatment received (Table 3). There was no significant correlation between the GI symptoms and the need for intensive care or mechanical ventilation, mortality rate, and the length of stay in the intensive care unit and medical floor (Table 4).

## Discussion

The COVID-19 pandemic continues to pose a significant threat to health worldwide and in our country. Although it is known that COVID-19 affects the GI system, the prevalence of GI symptoms accompanying the disease varies, and how GI involvement affects the prognosis remains controversial. In addition, it is thought that COVID-19 patients may apply to a healthcare facility showing only GI symptoms, and awareness about this will reduce the risk of infection. Our study shows our country’s data by examining the presence of GI symptoms and their effect on the prognosis in patients hospitalized with COVID-19 diagnosis.

Coronavirus disease 2019 (COVID-19) affects not only the respiratory system but also the digestive system.^5^ Studies show viral nucleic acid in stool samples of around 53.4% of patients.^5,15^ It was reported that although the throat and nasal swabs tested negative for COVID-19, the stool test was positive in some patients, and it was shown that PCR remained positive in rectal shedding for up to 10 weeks after the onset of symptom.^5,16^ There are various theories about the mechanisms of GI involvement in COVID-19 as it has not been fully comprehended. The affinity of the virus toward the angiotensin-converting enzyme 2 receptors of the epithelium of the GI system, the virus damaging the digestive system by triggering an inflammatory cascade, the virus itself changing the intestinal flora and increasing intestinal permeability, various pathophysiological mechanisms such as bowel ischemia in case of hypoxia in severe disease were found to be responsible for causing GI symptoms.^6,7,9,17^

The prevalence of the GI symptoms in COVID-19 ranges from 3% to 79%.^9^ In our study, we found the rate of accompanying GI symptoms (diarrhea, nausea, vomiting, abdominal pain, and the loss of taste) at the time of diagnosis as 12.1% in patients, who were hospitalized with a COVID-19 diagnosis. In a retrospective study by Chen et al^18^ that involved 209 patients in a single center in China, the presence of GI symptoms in COVID-19 patients at the time of admission was found to be 3%. The reason why the rate of GI symptoms accompanying COVID-19 was found to be lower than both our study and other studies in the current literature may be due to the fact that only diarrhea and nausea–vomiting were considered as GI symptoms in this study, and other GI symptoms such as abdominal pain and loss of taste were not questioned.^18^ The main reason for the heterogeneity of studies is that each study examined different GI symptoms. Some studies examined only diarrhea and nausea–vomiting as GI symptoms while others considered nonspecific symptoms such as the loss of taste and appetite as a GI symptom as well. For example, Pan et al^17^ found the rate of GI symptoms to be 50.5% in their multicenter study that included 204 patients. Still, they found the rate of specific GI symptoms (diarrhea, nausea, and abdominal pain) as 18.6% when they excluded the loss of appetite from their analysis. Besides, 3% of the patients in the study showed GI symptoms only without any respiratory symptoms. This finding shows that patients may show atypical symptoms when they apply to a hospital. In another study conducted in Wuhan, China, the rate of GI symptoms was found to be as high as 79.1%, and the patients’ GI symptoms were evaluated at the time of diagnosis and the following 10 days.^19^ In this study, both specific and nonspecific GI symptoms were examined and detected diarrhea in 49.5%, loss of appetite in 50.2%, vomiting in 29.4%, nausea in 15.9%, and abdominal pain in 6% of the patients. The reason why the rate of GI symptoms is high in this study may be that it included all GI symptoms and asked about the presence of GI symptoms both at the first admission and during the follow-up of the patients. Similarly, Lin et al^4^ found GI symptoms in 61% of a total of 95 COVID-19 patients, with a rate of 11.6% at the time of admission and 49.5% during follow-up at the hospital. They reported that GI symptoms that developed during patients’ stay at the hospital may be the result of the medication used.^4^ In a retrospective study by Jin et al^1^ that included 651 patients, which reported similar GI symptoms to our study, they found the rate of at least 1 specific GI symptom such as nausea, vomiting, or diarrhea at the time of diagnosis to be 11.4% in COVID-19 patients. In our country, in a study that included only 25 hemodialysis patients, nausea–vomiting or diarrhea was found in 10 patients (40%) at the time of diagnosis, and these patients did not report taste loss or abdominal pain.^20^ The reason why the rate of the GI symptoms in that study, which included fewer patients, was higher than ours may be the fact that the GI symptoms can occur due to chronic renal failure and hemodialysis treatments. As can be seen, the rate of the GI symptoms accompanying COVID-19 varies in studies. This heterogeneity may be the result of inpatient or outpatient treatment, diverse clinical manifestations (accompanying diseases, medications used), different times of symptom questions, and the variety in the GI symptoms that were chosen to be evaluated.

The GI symptoms in COVID-19 vary significantly including nausea, vomiting, abdominal pain, diarrhea, loss of taste, and bleeding, and the most common GI symptom that urges the patient to apply to a hospital varies.^9^ In our study, the most common GI symptoms were nausea and vomiting with a rate of 7.1%, followed by diarrhea as the second most common GI symptom with a rate of 2.8%. In the literature, diarrhea is the most common GI symptom in COVID-19 patients with a rate of 2%-50%.^8,9^ The prevalence of diarrhea in COVID-19 patients was 37% in a study conducted by Luo et al^21^, while another study conducted in Hubei, China, revealed it as 18.1%.^10^ This difference in prevalence rates results from the fact that the studies did not use a common definition for diarrhea. Furthermore, as the awareness about GI symptoms increased in the latter days of the pandemic, GI symptoms were questioned more often, causing its prevalence to increase in more recent studies. In addition, the prevalence of diarrhea varies in COVID-19 patients, who had diarrhea at the time of admission, hospitalization, and during the follow-up period. Supporting this finding, in a study conducted by Lin et al^4^ that included 95 patients with COVID-19, 5.3% of the patients had diarrhea at the time of admission and 18.9% of hospitalized patients had diarrhea during follow-up at the hospital. Some COVID-19 patients may not have diarrhea at the time of admission. If diarrhea occurs later during hospitalization, it is difficult to say whether it is due to the direct cytopathic effect of the SARS-Cov-2 virus or the indirect effect of antiviral treatments, antibiotics, and impaired intestinal flora. We believe that the lower prevalence of diarrhea in our study is due to the fact that we addressed GI symptoms only at the time of admission, and we used a clearer definition of diarrhea. The prevalence of nausea (1%-29.4%), vomiting (3.6% -66.7%), and abdominal pain (2.2% -6%) varies in studies.^9^ As we evaluated patients’ data in the early days of the pandemic, when the awareness of GI symptoms was much lower, the prevalence of GI symptoms can be lower than the rates reported by other studies in the literature. Although the loss of appetite is a common complaint in COVID-19 patients, its prevalence varies between 12.2% and 50.2% in studies; and it is usually accompanied by other symptoms, although some studies report that it is more common than diarrhea.^8^ The loss of appetite can be caused by intense inflammation, hypoxia, possible liver damage, depression or occur as a side effect of COVID-19 medication, and it is not possible to evaluate it objectively. Therefore, we did not consider the loss of appetite as a specific GI symptom and excluded it in our analysis of the prevalence of GI symptoms.

The studies in the literature show that there is no statistically significant difference in terms of demographic features between the patients with and without GI symptoms, similar to our study.^1,4,17^ In a study by Zhou et al^10^ where a total of 254 patients were divided into 2 groups, healthcare professionals and others, no correlation between the presence of GI symptoms and age was found, but it was seen that these complaints were more common, especially in female patients who were not healthcare professionals.^10^ In our study, unlike others, the mean age of patients with GI symptoms was lower, but we found no difference between genders, and this outcome was in line with the literature.^1,4,7,17^ Contrary to studies showing that chronic diseases do not affect the prevalence of GI symptoms^4,7^ a study conducted in China showed that GI symptoms were more common in patients with chronic liver disease.^1^ As our study did not include patients with a history of chronic liver disease, we could not comment on this issue. In our study, however, we found a lower prevalence of GI symptoms in patients with any comorbid condition. This may be due to the fact that patients do not care about GI symptoms at the beginning of the pandemic due to their accompanying comorbidities and are less likely to apply to the hospital.

The most common complaint by our patients when they first applied to the hospital was fever, and we did not find a correlation between fever and GI symptoms. However, a study conducted by Jin et al^4^ attribute fatigue, shortness of breath, and headache, which are more common complaints in patients with GI symptoms, to high fever and electrolyte disorder, which are also common in such patients.^1^ In a multi-center cohort study by Redd et al^22^ that was conducted in the United States, although the loss of taste was not considered a GI symptom, a significant correlation was found between symptoms of malaise, myalgia, dyspnea, sore throat, loss of taste and smell, and GI symptom.^22^ Although the loss of taste and smell in particular were associated with nausea and anorexia, the exact cause of this correlation was not determined. In our study, we could not find any differences in terms of accompanying symptoms other than loss of smell in patients with GI symptoms.

A study presenting clinical characteristics of COVID-19 patients with GI symptoms also showed that AST values of such patients were also higher than those without GI symptoms in addition to patients with accompanying liver disease having a higher rate of GI symptoms.^1^ A multicenter study conducted in China showed that GI symptoms became more significant as the severity of the disease increased. In parallel, more abnormalities in laboratory results were found in these patients such as elevated AST and ALT levels, decreased monocyte count, and increased prothrombin time. In particular, the high levels in liver function tests were attributed to the antimicrobial therapy the inpatients received.^17^ On the other hand, we did not find a correlation between GI symptoms and laboratory parameters except for D-dimer results of the COVID-19 patients we treated in the pandemic unit. The reason for the low D-dimer level in patients with GI symptoms may be due to the low mean age of this patient group because several studies have shown that D-dimer levels increase with age.^23^

The clinical significance of the presence of GI symptoms in COVID-19 patients remains unclear in the literature, and the results of studies are contradictory. In our study, no significant relationship was found between the presence of GI symptoms and the severity of the disease, the treatment received, risk of ARDS and septic shock, the need for ICU, the need for mechanical ventilation, mortality rate, and length of hospitalization in the medical floor and the intensive care unit. The study by Jin et al^1^ suggesting that the GI symptoms negatively affect the prognosis of the disease, found that patients suffering from the chronic liver disease had more GI symptoms, and the disease severity, need for mechanical ventilation, risk of liver damage, risk of ARDS, and the need for intensive care were higher in patients with GI symptoms.^1^ Contrary to this study, most of the publications on this subject showed that there is no correlation between the presence of GI symptoms and the clinical course. In a multicenter study that included 204 patients with pneumonia due to COVID-19 receiving treatment on a medical floor or intensive care unit, it was shown that GI symptoms became more significant as the disease got more severe, but there was no correlation between GI symptoms and length of hospitalization, the need for intensive care, or mortality.^17^ In another retrospective study including 150 hospitalized patients in the United States, the researchers found no difference between mortality, length of hospitalization, and the need for mechanical ventilation in the patient groups with and without GI symptoms, although the rate of pneumonia was higher in patients without GI symptoms.^7^ In this study, the clinical course may not be adversely affected since GI symptoms were not significantly higher in patients with more comorbidities, multi-medication use, and typical COVID-19 symptoms. Contrary to all these, some studies suggest that the GI symptoms in COVID-19 patients may positively affect the prognosis of the disease. In a retrospective study by Nobel et al^24^ the accompanying GI symptoms were associated with a lower rate of hospitalization in the ICU and a lower mortality rate although GI symptoms were more common in patients who tested positive for COVID-19. The authors considered this result as a milder form of COVID-19 in patients with a clinical picture accompanied by GI symptoms. Similarly, in a prospective single-center study conducted by Schettino et al^25^ in Italy, they examined 190 patients and found that 69% of the patients had at least one GI symptom. They also found that the mortality rate was lower in patients with GI symptoms. The researchers think that the local inflammatory response triggered after viral infection changes intestinal microbiota, and this immune regulation has a positive effect on the prognosis of the disease.

As can be seen, reports about COVID-19 and its effects on the GI system vary. In our country, in a retrospective study by Avcı et al^26^ that included 110 patients, the researchers found the rate of GI symptoms in COVID-19 patients at the time of admission to be 11.8%, which is close to the result of our study. The researchers included only the patients, who had a positive RT-PCR test result from nasopharyngeal swabs, and they described nausea, vomiting, abdominal pain, and diarrhea as the GI symptoms. Since the numbers were insufficient in their study, Avcı et al^26^ could not determine a correlation between admission to the ICU, mechanical ventilation, and mortality, but they reported that the presence of GI symptoms had a high predictive value in terms of severity of the disease. In addition, they found that LDH, CRP, ferritin, fibrinogen, and D-dimer levels were higher in patients with GI symptoms.

Our study had some limitations. First of all, our study is a retrospective single-center study, and there are no generally accepted definition criteria for the definition of symptoms except for diarrhea. Secondly, our results may not apply to all categories of COVID-19 patients since this study was conducted with hospitalized patients only. Furthermore, we could not confirm the GI involvement by rectal swabbing or taking intestinal tissue samples for RT-PCR. Notwithstanding these limitations, we did not overlook symptomatic patients whose swab samples tested negative as our study included not only the patients whose nasal swabs tested positive but also those with typical COVID-19 findings clinically and in thorax CT.

In conclusion, we did not find a correlation between the accompanying GI symptoms and the severity and prognosis of COVID-19. Our study clearly demonstrates the prevalence and severity of the GI symptoms in COVID-19 patients and the effect of the disease on the clinical outcomes. It must be kept in mind that COVID-19 disease can manifest itself with non-respiratory symptoms such as GI symptoms, and such patients should be examined being cautious about COVID-19. More multicenter and multinational studies involving more COVID-19 patients are needed to determine the prevalence of the accompanying GI symptoms, diagnosed based on objective diagnostic criteria, and to determine the correlation between this association and the prognosis of the disease.

## Figures and Tables

**Table 1.** Demographics and Clinical Features of Coronavirus Disease 2019

	**All COVID-19 (n = 322)**	**GI Symptoms** **(n = 39)**	**No GI Symptoms (n = 283)**	***P***
**Age (years)**	61.02 ± 20.63 (46-79)	53.72 ± 19.71 (35-75)	62.03 ± 20.58 (47-80)	**.018**
**Gender (male)**	159 (49.4)	21 (53.8)	138 (48.8)	.552
**Smoker (n = 147)**				
No exposure	80 (54.4)	15 (62.5)	65 (52.8)	.137
Quit smoking	48 (32.7)	4 (16.7)	44 (35.8)	
Current smoker	19 (12.9)	5 (20.8)	14 (11.4)	
**Medical staff**	22 (6.8)	3 (7.7)	19 (6.7)	.738
**Immunosuppression**	31 (9.7)	2 (5.1)	29 (10.3)	.398
**Comorbidities**	220 (68.3)	19 (48.7)	201 (71)	**.005**
Chronic respiratory diseases	37 (11.5)	4 (10.3)	33 (11.7)	.999
Hypertension	144 (44.7)	15 (38.5)	129 (45.6)	.402
Heart disease	42 (13)	3 (7.7)	39 (13.8)	.290
Chronic renal disease	25 (7.8)	2 (5.1)	23 (8.1)	.752
Diabetes mellitus	53 (16.5)	4 (10.3)	49 (17.3)	.265
Cerebrovascular disease	13 (4)	1 (2.6)	12 (4.2)	.999
Cancer	25 (7.8)	1 (2.6)	24 (8.5)	.335
**Medications**	202 (64.7)	18 (48.6)	184 (66.9)	**.029**
ACEIs/ARBs	71 (22)	7 (17.9)	64 (22.6)	.510
OADs/İnsülin	46 (14.3)	2 (5.1)	44 (15.5)	081
Others	137 (42.5)	11 (28.2)	126 (44.5)	.053
**Symptoms**	291 (90.4)	39 (100)	252 (89)	**.021**
Fever	159 (49.4)	23 (59)	136 (48.1)	.201
Cough	134 (41.6)	19 (48.7)	115 (40.6)	.337
Expectoration	2 (0.6)	0 (0)	2 (0.7)	.999
Dyspnea	85 (26.4)	8 (20.5)	77 (27.2)	.374
Fatigue	64 (19.9)	12 (30.8)	52 (18.4)	.069
Loss of smell	7 (2.2)	5 (12.8)	2 (0.7)	**<.001**
Myalgia	12 (3.7)	1 (2.6)	11 (3.9)	.999
Sore throat	32(9.9)	3 (7.7)	29 (10.2)	.780
Rinore	10 (3.1)	2 (5.1)	8 (2.8)	.347
Headache	20 (6.2)	5 (12.8)	15 (5.3)	.079

Data are expressed as mean ± SD, median (IQR) and n (%).

SD, standard deviation; IQR; interquartile range; GI, gastrointestinal; ACEIs/ARBs, angiotensin-converting enzyme inhibitors/angiotensin receptor blockers; OADs, oral antidiabetic drugs.

**Table 2.** Laboratory and Radiology Findings of COVID-19

	**All COVID-19 (n = 322)**	**GI Symptoms (n = 39)**	**No GI Symptoms (n = 283)**	***P***
**Complete blood count**				
Hb (g/dL)	13.21 ± 1.83	13.61 ± 1.67	13.15 ± 1.85	.148
Leucocytes (µL)	6300 (4800-8500)	5900 (4500-8000)	6300 (4800-8500)	.315
Neutrophils (µL)	4224 (3000-6371)	4100 (2940-6162)	4280 (3002-6399.5)	.533
Lymphocytes (µL)	1200 (858-1700)	1303 (891-1700)	1195.5 (822.5-1681)	.328
NLR	3.34 (2.2-5.99)	2.81 (1.92-5.35)	3.38 (2.23-6.01)	.248
**Coagulation function**				
d-dimer (µg/mL feu)	0.7 (0.4-1.3)	0.5 (0.4-0.9)	0.7 (0.4-1.5)	**.043**
**Blood biochemistry**				
Creatinine (mg/dL)	0.84 (0.65-1.06)	0.86 (0.61-1)	0.84 (0.65-1.06)	.584
AST (U/L)	28 (22-41)	30 (23-43)	27 (21-40)	.359
ALT (U/L)	22 (13-35)	24 (16-40)	21 (13-34)	.151
Albumin (g/dL)	3.8 ± 0.6	3.7 ± 0.4	3.8 ± 0.6	.676
LDH (U/L)	231 (183-320)	219.5 (179-320)	232 (183-327)	.652
Troponin (ng/L)	6 (5.5-14.2)	5.5 (5.5-9.7)	6.1 (5.5-14.7)	.127
Ferritin (ng/mL)	155.3 (64.4-340.9)	145.35 (69.45-332.75)	161 (62.9-341.5)	.821
**Infection-related biomarkers**				
CRP ( mg/L)	27.7 (10.6-81.4)	19.9 (10.8-82.3)	28 (10.4-81)	.962
Procalcitonin (ng/mL)	0.05 (0.03-0.1)	0.05 (0.03-0.08)	0.05 (0.03-0.1)	.663
Radiology				
Classical/probable COVID-19	194 (60.2)	28 (71.8)	166 (58.7)	.201
Indeterminate for COVID-19	40 (12.4)	6 (15.4)	34 (12)	
Non COVID-19 Features	32 (9,9)	2 (5.1)	30 (10.6)	
Normal	56 (17.4)	3 (7.7)	53 (18.7)	

Data are expressed as mean ± SD, median (IQR) and n (%).

COVID-19, coronavirus disease 2019; SD, standard deviation; IQR; interquartile range; GI, gastrointestinal; Hb, hemoglobin; NLR, neutrophil to lymphocyte ratio; AST, aspartate aminotransferase; ALT, alanine aminotransferase; LDH, lactate dehydrogenase; CRP, C-reactive protein.

**Table 3.** Treatment in Patients with COVID-19

	**All COVID-19 (n = 322)**	**GI Symptoms (n = 39)**	**No GI Symptoms (n = 283)**	***P***
Hydroxychloroquine	277 (86)	36 (92.3)	241 (85.2)	.227
Oseltamivir	144 (44.7)	21 (53.8)	123 (43.5)	.221
Azithromycin	60 (18.6)	7 (17.9)	53 (18.7)	.907
Fluoroquinolones	105 (32.6)	12 (30.8)	93 (32.9)	.794
Beta-lactamase inhibitors	82 (25.5)	7 (17.9)	75 (26.5)	.250
Favipravir	50 (15.5)	7 (17.9)	43 (15.2)	.656

Data are expressed as n (%).

COVID-19, coronavirus disease 2019; GI, gastrointestinal.

**Table 4.** Complications and Severity in Patients with COVID-19

	**All COVID-19 (n = 322)**	**GI Symptoms** **(n = 39)**	**No GI Symptoms (n = 283)**	***P***
Clinical classification				
Mild	56 (17.4)	3 (7.7)	53 (18.7)	.207
Moderate	205 (63.7)	29 (74.4)	176 (62.2)	
Severe	23 (7.1)	4 (10.3)	19 (6.7)	
Critical	38 (11.8)	3 (7.7)	35 (12.4)	
Complication				
ARDS	28 (8.7)	3 (7.7)	25 (8.8)	.999
Shock	29 (9)	3 (7.7)	26 (9.2)	.999
Admission to ICU	38 (11.8)	3 (7.7)	35 (12.4)	.596
Mechanical ventilation	33 (10.2)	3 (7.7)	30 (10.6)	.780
Medical floor stay (day)	5 (3-9)	5 (2-8)	5 (3-9)	.562
ICU stay (day)	5 (2-9)	6 (0-20)	4.5 (2-9)	.937
In-hospital deaths	32 (9.9)	2 (5.1)	30 (10.6)	.397

Data are expressed as median (IQR) and n (%).

COVID-19, coronavirus disease 2019; IQR, interquartile range; GI, gastrointestinal; ARDS, acute respiratory distress syndrome; ICU, intensive care unit.
